# *In Vivo* Injection of Reversible Optically Controlled Short Interfering RNA into Japanese Medaka Embryos (*Oryzias latipes*) to Regulate Gene Silencing

**DOI:** 10.1021/acschembio.4c00290

**Published:** 2024-08-20

**Authors:** Makenzie Mateus, Matthew L. Hammill, Denina B. D. Simmons, Jean-Paul Desaulniers

**Affiliations:** Faculty of Science, Ontario Tech University, 2000 Simcoe Street North, Oshawa ON L1G 0C5, Canada

## Abstract

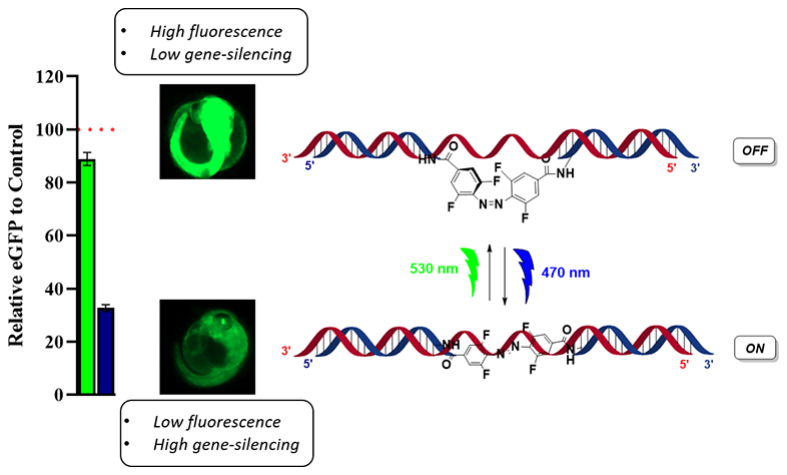

Photoswitchable *ortho*-functionalized tetrafluorinated azobenzene-modified siRNAs (F-azo-siRNAs) were synthesized using solid-phase phosphoramidite chemistry. The activity of an F-azo-siRNA targeting enhanced green fluorescence protein (eGFP) in transgenic (Tg) Japanese Medaka (*Oryzias latipes*) was reversibly photocontrolled with blue (470 nm) and green (530 nm) light, to activate and inactivate the siRNA, respectively. This study highlights the first reversible *in vivo* study with photoswitchable siRNA. Controlling siRNA function reversibly *in vivo* could open new opportunities for biotech research to better understand gene function and cellular mechanisms.

In 1998, Fire and co-workers reported the RNA interference (RNAi) pathway in *Caenorhabditis elegans*.^1^ This endogenous pathway uses double-stranded RNAs called short interfering RNAs (siRNAs) to control gene expression.^2^ Since then, siRNAs have been and continue to be an indispensable tool to study gene function in biological systems.^3^

The photoresponsive field has grown rapidly over the years because it is an excellent research tool to understand biomolecular mechanisms that govern complex biological systems.^4,5^ These tools are useful when applied to transparent model organisms. Many clever photocontrolled gene silencing systems with oligonucleotides have been developed.^6−9^ However, one common theme is that these designs are not reversible once photoactivated. Our lab has been exploring the effect and impact of a reversible photoswitchable molecule called azobenzene when embedded within siRNAs.^10,11^ Azobenzene can reversibly photoisomerize between *trans* and *cis* with different wavelengths of light.^12,13^ In our most recent work, we designed a synthesis for an *ortho*-functionalized tetrafluorinated azobenzene phosphoramidite and synthesized an siRNA bearing this modification within the central region.^14^ This siRNA could be reversibly controlled with green (530 nm) and blue light (470 nm) for up to 72 h within *in vitro* cell line experiments (see Figure 1). The objective of this study was to examine the effect of an F-azo-siRNA over a greater length of time and within a more appropriate *in vivo* context. This paper describes the use of a photoswitchable F-azo-siRNA in transgenic Japanese Medaka embryos over a 10-day span. Japanese medaka (*Oryzias latipes*) are small freshwater teleost fish that have desirable features for use as a vertebrate model.^15^ Their embryos are attractive as a model *in vivo* system because they have a transparent egg chorion and rapid growth stages and there are a variety of useful strains and a sequenced genome.

**Figure 1:**
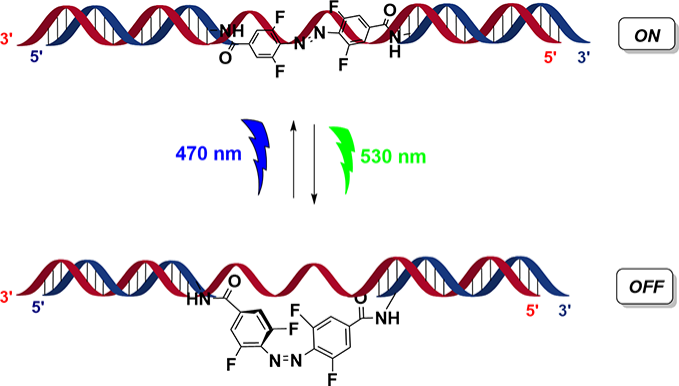
An *ortho*-functionalized tetrafluorinated azobenzene embedded within the siRNA duplex, showing an active ON form (*trans*) that can be converted to an inactive OFF form (*cis*) using green light. The process is reversible with blue light treatment.

To highlight why it would be useful to have a reversible siRNA tool *in vivo* to control activity, a study from Alnylam highlighted that a GalNAc-conjugated siRNA exhibited rat hepatotoxicity.^16^ The hepatotoxicity could be attenuated with their technology called REVERSIR, which is a short chemically modified oligonucleotide that competes for the RNA-induced-silencing-complex (RISC)-loaded guide strand, thus inactivating the siRNA activity. Although this technology can inhibit the activity of an already deployed siRNA, it requires an additional injection and is not reversible.

SiRNAs are an indispensable tool for molecular biology. For example, loss-of-function siRNA screens have been used to identify key proteins in metabolic pathways.^17,18^ Being able to reversibly control the *in vivo* function of an siRNA in molecular biology would enhance our understanding of the biomolecular mechanisms governing these complex biological pathways.

Table 1 highlights the siRNA strands that were synthesized and injected into the Japanese Medaka embryos. We selected a previously studied siRNA sequence that has been shown well to silence eGFP effectively in Japanese Medaka.^19^ We synthesized a red-shifted *ortho*-functionalized tetrafluorinated azobenzene-containing siRNA, F-siRNA-G1, which includes the tetrafluorinated azobenzene moiety, replacing two bases between positions 10 and 11 on the antisense strand, counting from the 5′-end.^14^ We also synthesized a scrambled control sequence that also contained a red-shifted tetrafluorinated azobenzene-containing siRNA (SCR-F-siRNA-G2) bearing the tetrafluorinated azobenzene moiety between the same positions (10 and 11 on the antisense strand) as the target sequence. As predicted, F-siRNA-G1 exhibited classic A-form duplex as characterized by circular dichroism (Supplementary Figure S-2).^20^ In addition, to confirm if F-siRNA-G1 would respond to green light, we incubated the antisense strand to green light. As expected, HPLC analysis shows a shorter retention time, indicating that the *cis* conformer is formed after green light exposure (Supplementary Figure S-1).

**Table 1:** SiRNAs That Were Tested on Transgenic Japanese Medaka Embryos Expressing eGFP^a^

name	siRNA sequence	target
wt-siRNA-G1	3′-dTdTGUUCGACUGGGACUUCAAG-5′ (s)	eGFP
	5′-CAAGCUGACCCUGAAGUUCdTdT-3′ (as)	
F-siRNA-G1	3′-dTdTGUUCGACUGGGACUUCAAG-5′ (s)	eGFP
	5′-CAAGCUGACF-AzoUGAAGUUCdTdT-3′ (as)	
SCR-F-siRNA-G2	3′-dTdTGUACGUCGUGAGCUUACAG-5′ (s)	N/A
	5′-CAUGCAGCAF-AzoCGAAUGUCdTdT-3′ (as)	

a Wt-siRNA-G1 reflects the unmodified sequence used to silence eGFP, while F-siRNA-G1 represents the tetrafluorinated azobenzene-modified siRNA. SCR-F-siRNA-G2 represents a scrambled sequence used to act as a control. Incorporation of the tetrafluorinated azobenzene modification is denoted as F-Azo. (s) and (as) correspond to sense and antisense, respectively.

A transgenic strain of Japanese medaka (d-rR-Tg(eGFP)) was purchased from the National BioResource Project (NBRP) in Japan and maintained and bred in the Aquatic Facility at Ontario Tech University in accordance with a breeding protocol approved by the Ontario Tech Animal Care Committee. This transgenic strain allows for the silencing of eGFP to be easily and efficiently examined after injection with siRNAs in single cell embryos. Once embryos were collected, they were injected with siRNAs at the one-cell stage to assess their gene-silencing capability against eGFP over 10 days. Fluorescence readings were collected every 24 h post injection (hpi).

Eight nanograms of wt-siRNA-G1 was injected, and these embryos were kept in the dark for the duration of the experiment. As shown in Figure 2, a decrease in eGFP of approximately 23% was observed starting at 24 hpi. We observed a steady decline in the fluorescence until 120 hpi, after which fluorescence started to progressively increase afterward. This was supported by the results of the ANOVA analysis, with a significant decrease in expression when comparing the 24–120 hpi embryos to noninjected control embryos denoted by the red dotted line. After this, we observed a significant increase in fluorescence from 120 hpi to 144 hpi (33% to 60%), suggesting that the siRNA has been used completely and/or has been degraded by a nuclease enzyme. The trends associated with this experiment closely resemble the wt-siRNA-G1 data, which is expected, as we have shown similar silencing levels with identically positioned tetrafluorinated azobenzene-embedded siRNAs within an *in vitro* cell culture line.^14^

**Figure 2:**
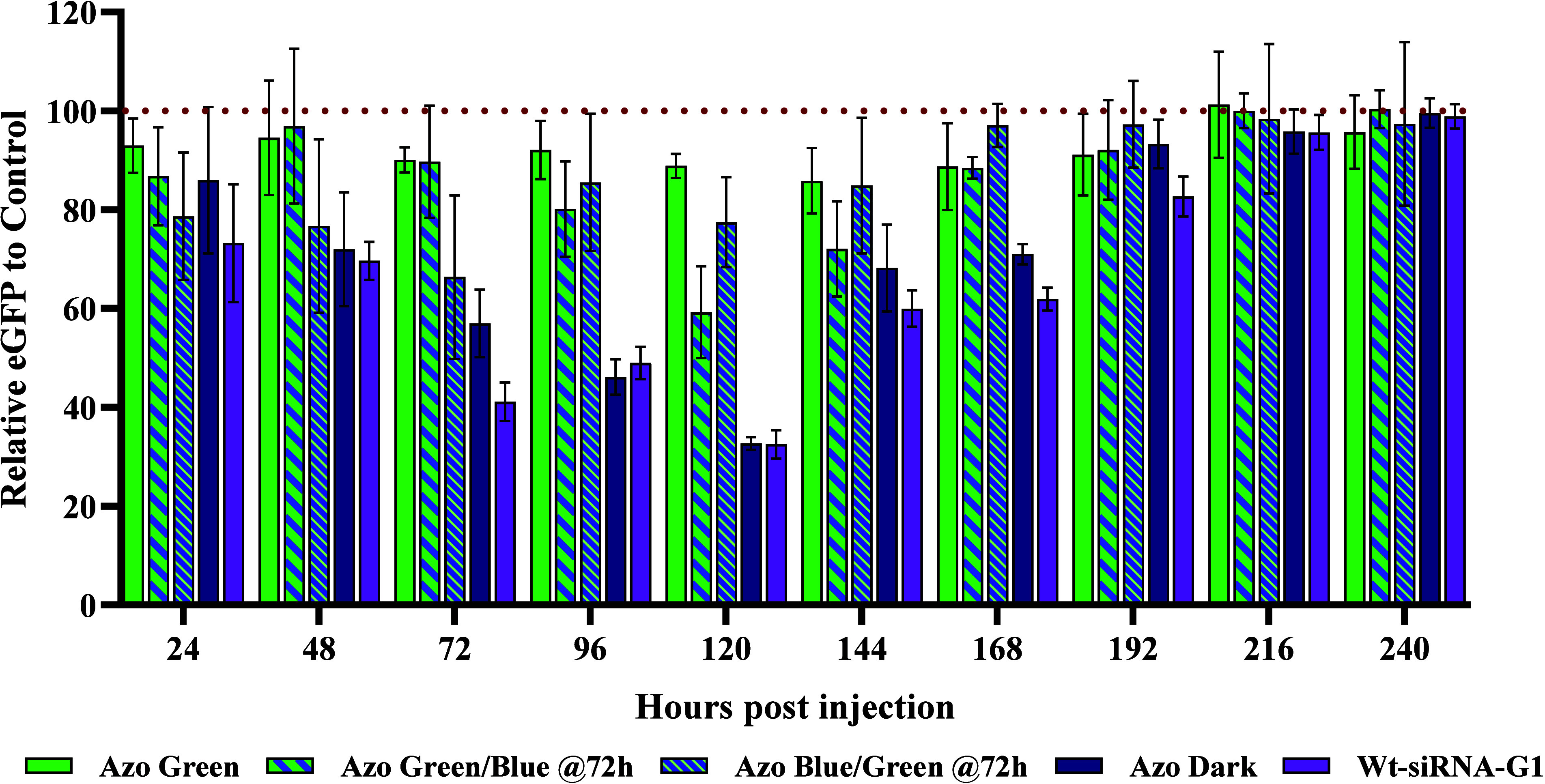
Normalized enhanced green fluorescent protein (eGFP) expression for F-siRNA-G1 and wt-siRNA-G1 at 8 ng in Japanese medaka embryos 24–240 hpi. Azo Green corresponds to embryos being injected with F-siRNA-G1 and being exposed to green 530 nm wavelength light at 0 hpi every 24 h (*n* = 5). Azo Green/Blue @72h corresponds to embryos treated with F-siRNA-G1 and exposing them at 0, 24, and 48 hpi to green light; however, at 72 hpi the eggs were exposed to 470 nm blue wavelength light every 24 h (*n* = 7). Azo Blue/Green @72h corresponds to embryos treated with F-siRNA-G1 and exposing them to blue light at 0, 24, and 48 hpi; however, at 72 hpi, the eggs were exposed to green light every 24 h (*n* = 8). Azo Dark corresponds to embryos being injected with F-siRNA-G1 kept in the dark (*n* = 7). Wt-siRNA-G1 corresponds to embryos being injected with wt-siRNA-G1 (*n* = 12).

Now that we have established that both the unmodified (wt-siRNA-G1) and the tetrafluorinated azobenzene-modified siRNA (F-siRNA-G1) exhibit time-dependent gene silencing after several days, we then wanted to assess if we could keep the F-siRNA-G1 in an inactive form with the use of green light (530 nm). In the next set of experiments, immediately after injection, the embryos were incubated with green light for 1 h and then kept in the dark. After each fluorescence reading measurement every 24 hpi, the embryos were exposed to some natural light. Thus, the embryos were re-exposed to the same duration and intensity of green light before being placed back in the dark. As shown in Figure 2, there was no statistical variance in the green-light treated embryos in the 24-to-240-h time period for the 8 ng of injected F-siRNA-G1 embryos. This observation suggests that the azobenzene molecular switch was in the off position due to green light exposure for the duration of this experiment, and thus minimal siRNA activity occurred (Figure 2).

With the confirmation that the green light treatment of the F-siRNA-G1 remains inactive, we then tested the ability to activate F-siRNA-G1 with the treatment of blue light (470 nm). In this experiment, the F-siRNA-G1 was kept inactive with green light treatment (1 h of green light every 24 h) up to 72 h. At the 72-h time point, the embryos were exposed to blue light every 24 hpi to reactivate the F-siRNA-G1 to the *trans* conformer. Fluorescence data measured after the 72 hpi indicates that a mild drop in fluorescence occurred at 96 hpi, then a significant drop after 120 hpi, suggesting that the F-siRNA-G1 can be activated after 3 days of inactivity. After 120 hpi, fluorescence starts to increase, which is consistent with the other test conditions (Figure 2).

Next, the effect of inactivating the F-siRNA-G1 at 72 hpi was performed. After injection, the embryos were incubated with blue light (470 nm) and every 24 hpi after measurement readings up to the 72 h time point. As expected, and as indicated in Figure 2, the gene silencing with blue light treatment mimics the silencing observed (ca. 30% silencing) with F-siRNA-G1 with no light treatment used, for up to 72 h. At 72 hpi, the embryos were treated with green light every 24 h until the duration of the experiment was finished. From 96 hpi to 240 hpi, silencing is no longer occurring as indicated by the high levels of eGFP. This indicates that inactivation of the siRNA can occur after 72 h of silencing (Figure 2).

We conducted the same type of experiment with 4 ng injections of wt-siRNA-G1 and F-siRNA-G1 in Japanese medaka embryos and observed very similar effects to the 8 ng data (Figure 3). A slight reduction in potency was observed for the 4 ng data (ca. 50% silencing) at the greatest silencing point at 120 h, compared to the 8 ng data at the 120 hpi time point (ca. 60% silencing). This is exactly as expected since there is less siRNA available in the experiment. However, the same trends were observed with the different colored light additions. For all the eGFP silencing experiments described above, it should be noted that a delay in the decrease in fluorescence signal from eGFP after siRNA silencing occurs and is consistent with the half-life of GFP of approximately 26 h.^21^

**Figure 3:**
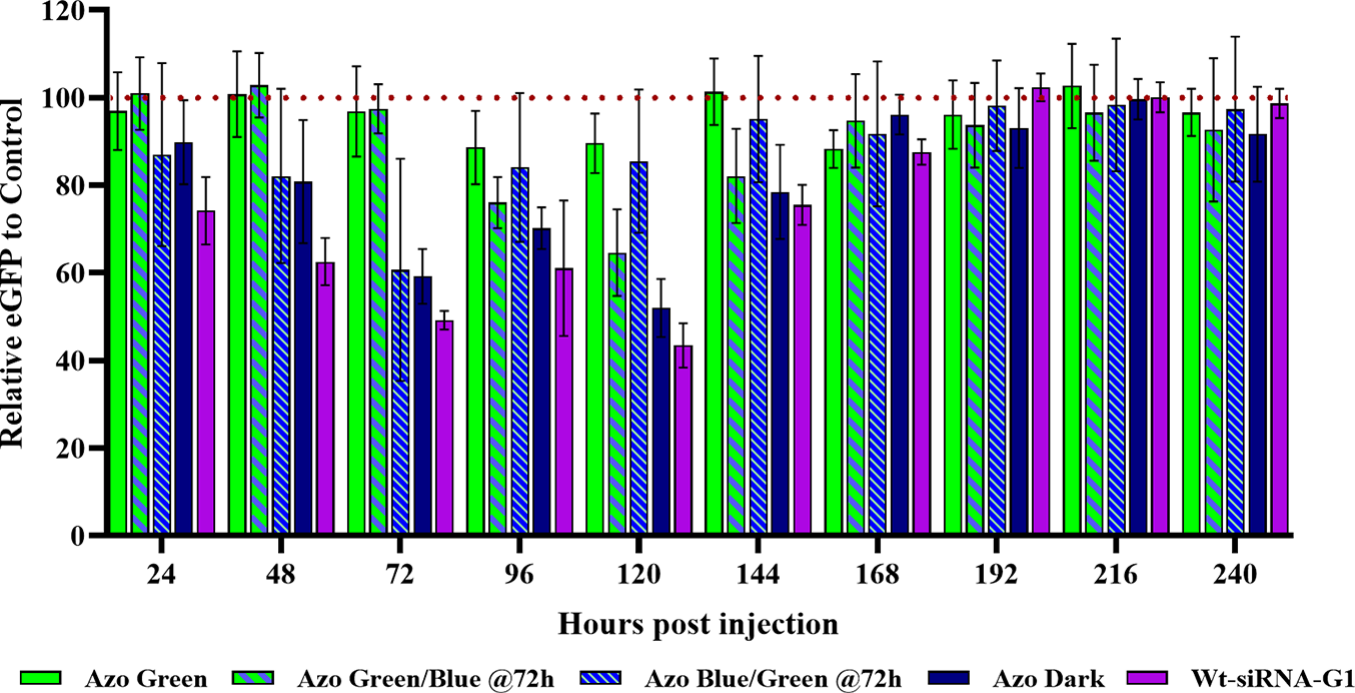
Normalized enhanced green fluorescent protein expression for F-siRNA-G1 and wt-siRNA-G1 at 4 ng in Japanese medaka embryos 24–240 hpi. Azo Green corresponds to the F-siRNA-G1 being exposed to green 530 nm wavelength light at 0 hpi every 24 h (*n* = 3). Azo Green/Blue @72h corresponds to embryos treated with F-siRNA-G1 and exposing them at 0, 24, and 48 hpi to green light; however, at 72 hpi, the eggs were exposed to 470 nm blue wavelength light every 24 h (*n* = 5). Azo Blue/Green @72h corresponds to embryos treated with F-siRNA-G1 and exposing them to blue light at 0, 24, and 48 hpi; however, at 72 hpi, the eggs were exposed to green light every 24 h (*n* = 8). Azo Dark corresponds to embryos being injected with F-siRNA-G2 and kept in the dark (*n* = 6). Wt-siRNA-G1 corresponds to embryos being injected with wt-siRNA-G1 (*n* = 9).

To ensure that the changes in fluorescent levels we were monitoring were indeed due to optical control of the F-siRNA-G1, we tested a scrambled nontargeting sequence (SCR-F-siRNA-G2) containing the tetrafluorinated azobenzene at the same position as F-siRNA-G1. As is observed in Figure 4, no significant changes in fluorescence are observed with SCR-F-siRNA-G2, in the presence of all the conditions used to test the photocontrol. Thus, this highlights that the photocontrolled changes in gene-silencing observed with F-siRNA-G1 are genuine and are target specific.

**Figure 4:**
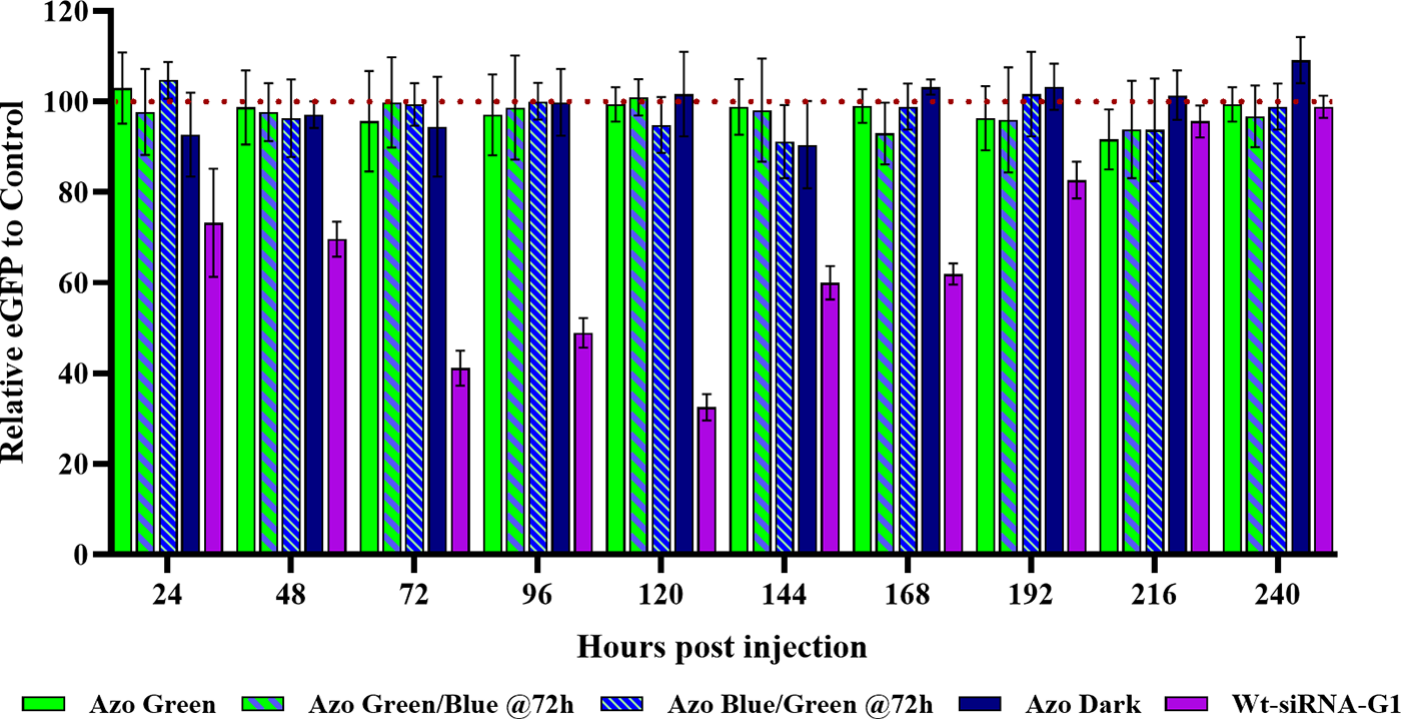
Normalized enhanced green fluorescent protein expression for SCR-F-siRNA-G2 and wt-siRNA-G1 at 8 ng in Japanese medaka embryos 24–240 hpi. Azo Green corresponds to embryos being injected with SCR-F-siRNA-G2 and being exposed to green 530 nm wavelength light at 0 hpi every 24 h (*n* = 5). Azo Green/Blue @ 72h corresponds to embroys treated with SCR-F-siRNA-G2 and exposing them at 0, 24, and 48 hpi to green light; however, at 72 hpi, the eggs were exposed to 470 nm blue wavelength light every 24 h (*n* = 5). Azo Blue/Green @72h corresponds to embroys treated with SCR-F-siRNA-G2 and exposing them at 0, 24, and 48 hpi to green light; however, at 72 hpi the eggs were exposed to green light every 24 h (*n* = 4). Azo Dark corresponds to embryos being injected with SCR-F-siRNA and kept in the dark (*n* = 4). Wt-siRNA-G1 corresponds to embryos being injected with wt-siRNA (*n* = 9).

To visualize the effect of gene-silencing on eGFP with the gene-silencing siRNAs, fluorescent embryo images were taken every 24 h for the duration of the experiment. The images from Figures 5–9 are select image representations of the medaka embryos used within the various conditions to generate Figures 2–4. These images show clear images of green fluorescence, which represents the expression of the eGFP (Figure 5). The embryo images of the eggs injected with 8 ng of F-siRNA-G1 with no light show a drop in fluorescence compared to the uninjected control from 48 to 144 hpi. From 168 to 240 hpi, the fluorescence levels increase. This correlates with the data from Figure 2.

**Figure 5:**
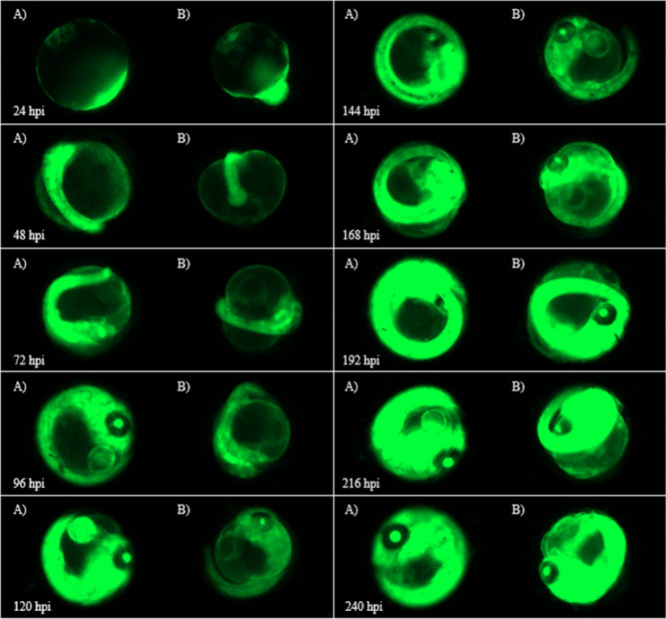
(A) Images of control noninjected Japanese Medaka embryos vs (B) embryos injected with 8 ng of F-siRNA-G1 kept in the dark (Azo Dark) from 24 to 240 hpi.

**Figure 6:**
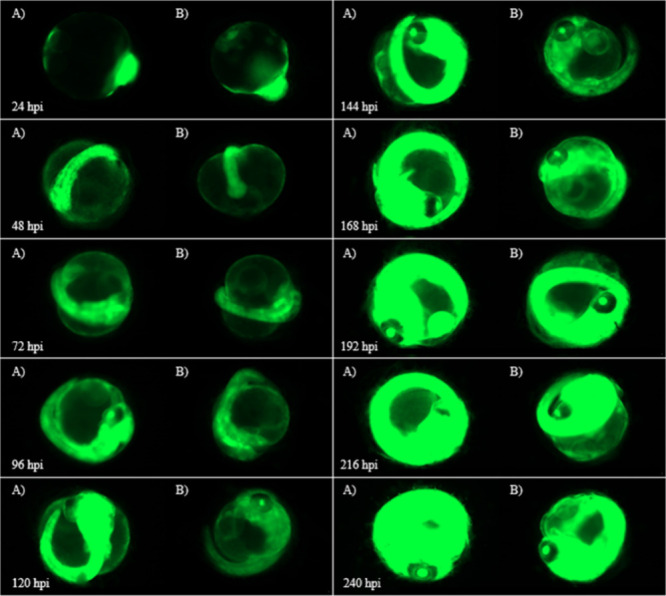
(A) Images of Japanese Medaka embryos injected with 8 ng of F-siRNA-G1 held under green light (Azo Green) vs (B) dark conditions (Azo Dark) from 24 to 240 hpi.

**Figure 7:**
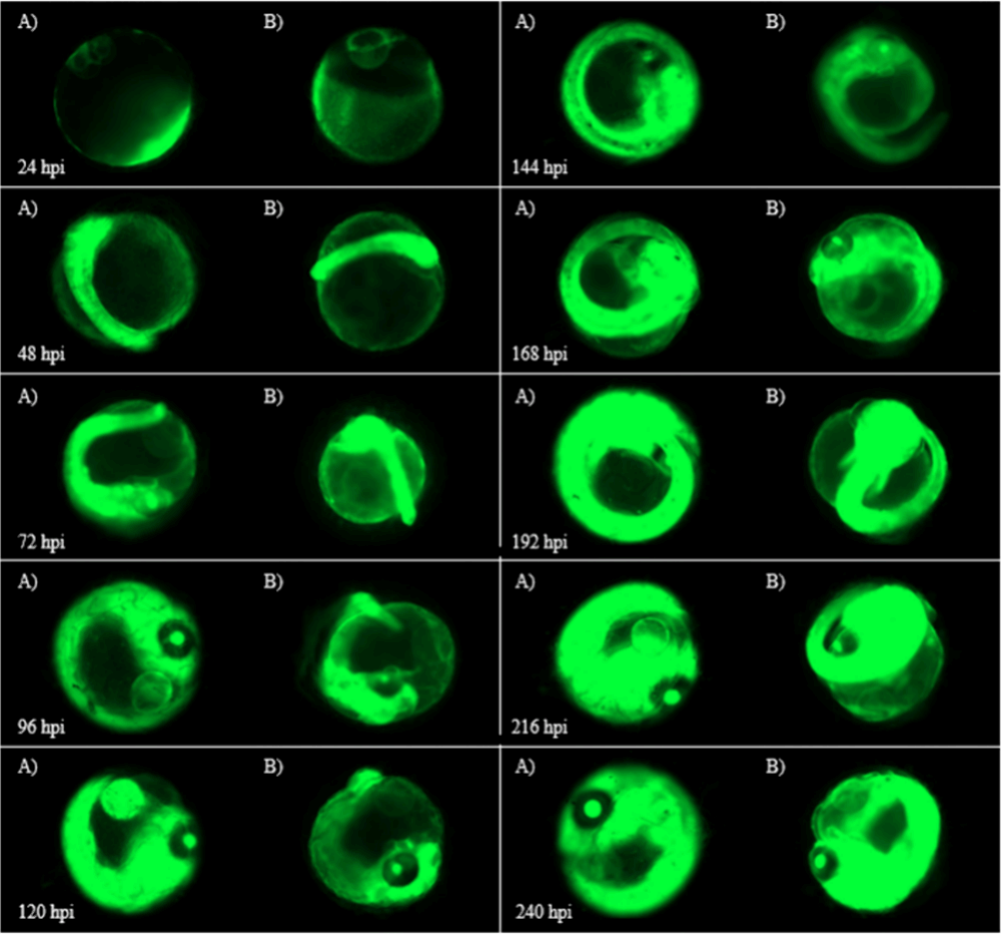
(A) Images of control noninjected Japanese Medaka embryos vs (B) embryos injected with 8 ng of F-siRNA-G1 held under green light for 1 at 0 hpi, 24 hpi, and 48 hpi then at 72 hpi held under blue light every 24 h until the 240 hpi time point (Azo Green/Blue @ 72 h).

**Figure 8:**
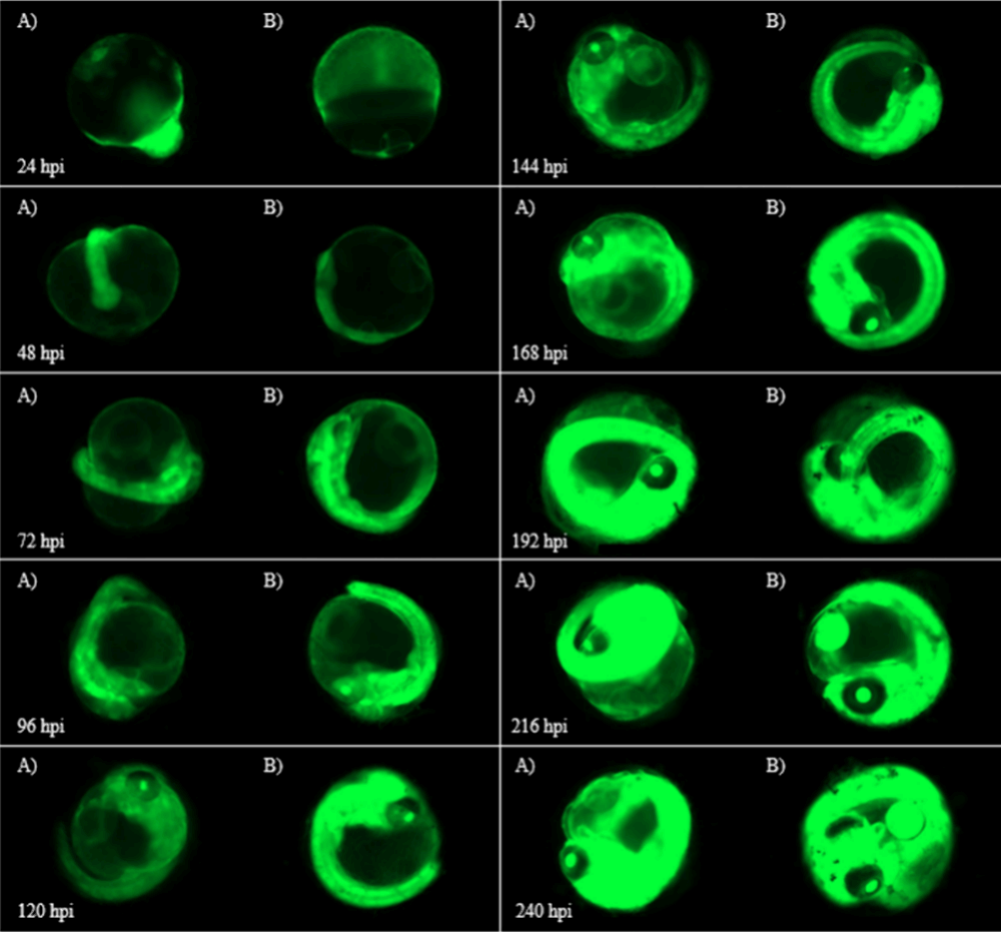
(A) Images of Azo Dark embryos vs (B) embryos injected with 8 ng of F-siRNA-G1 held under blue light for 1 at 0 hpi, 24 hpi, and 48 hpi then at 72 hpi held under green light every 24 until the 240 hpi time point. (Azo Blue Green @ 72 h).

**Figure 9:**
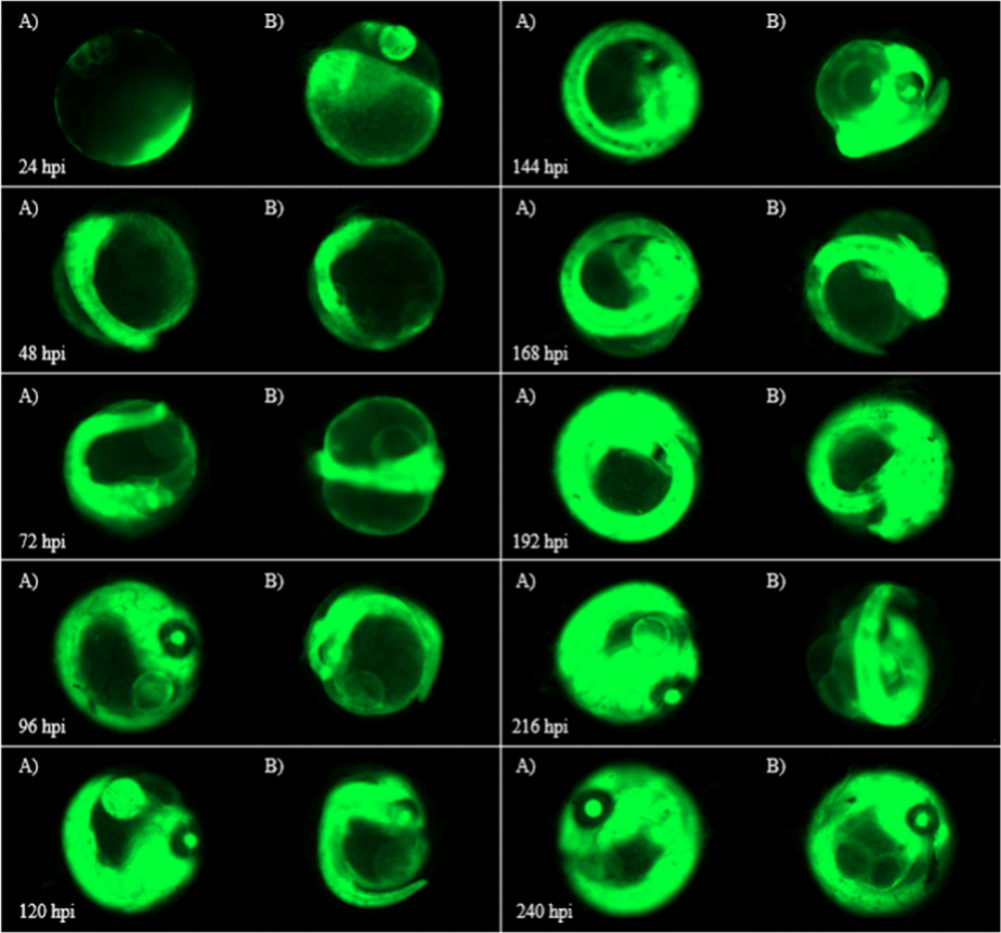
(A) Images of control noninjected Japanese Medaka embryos vs (B) embryos injected with 8 ng of SCR-F-siRNA-G2 held in the dark.

The fluorescent images in Figure 6 compare the effect of green light and no light on the F-siRNA-G1’s ability to silence in the embryo. As indicated, clear differences in fluorescence intensity are observed. When the embryo is treated with green light, a high intensity of fluorescence is observed, indicating that gene-silencing is being inhibited. In contrast, the dark treatment shows a much less intense fluorescence from 72 hpi to 168 hpi. This shows that the F-siRNA-G1 can be silenced in real time *in vivo*.

Figure 7 compares noninjected embryo fluorescence images to embryos treated with F-siRNA-G1 and inactivated with green light for 72 hpi, followed by blue light treatment until the 240 hpi time point. The images show clear differences at 120 and 144 hpi, which is expected. This is consistent with silencing data that it takes at least 48 h of active siRNA to be noticeable.

Figure 8 compares embryos treated with 8 ng of F-siRNA-G1 held under dark conditions and embryos treated with blue light for the first 72 h, followed by green light treatment until the 240 hpi time point. Clear differences are observed between 96 and 120 h, where the green treated embryos are showing increasing fluorescence compared to noninjected controls, thus indicating that inactivation of the F-siRNA-G1 has been successful with green light, even after the initial blue light exposure.

Finally, the embryo images of SCR-F-siRNA-G2 scrambled control azobenzene-modified siRNA show similar fluorescent levels to noninjected controls, highlighting that the scrambled sequence is not going through a gene-silencing mechanism. This highlights that target specificity of our F-siRNA-G1 is retained with the tetrafluorinated azobenzene siRNA (Figure 9).

Taking advantage of this system will allow for the further development of optically controlled reversible siRNAs that can target genes *in vivo*. For example, this could have a large impact on learning how to control the dose and potency of an siRNA as a tool for molecular biology, or to understand gene function. Furthermore, this study is also significant because, to the best of our knowledge, this is the first time that siRNAs have been successfully used in Japanese Medaka embryos. Thus, this opens the door for future siRNA function studies within this *in vivo* model system.

With respect to other fish studies, a study by Hofsteen et al. used an siRNA loss-of-function screen to identify that Alpha Protein Kinase 2 (ALPK2) promotes cardiogenesis in zebrafish (*Danio rerio*) and human pluripotent stem cells.^17^ Being able to control the silencing siRNA in an active and inactive state would be useful in further understanding the mechanisms of cardiogenesis.

Another study involving fish used rainbow trout (*Oncorhynchus mykiss*) as an *in vivo* model to test for the delivery and antiviral effects of siRNAs.^22^ The potential ability to reversibly control the function of these antiviral siRNAs with light would help to understand the molecular mechanism of action of candidate antiviral siRNAs.

In conclusion, we have developed a robust system to monitor the *in vivo* gene silencing of eGFP from a tetrafluorinated azobenzene containing siRNA in Japanese Medaka embryos. This system is robust and allows for the evaluation of photoswitchable siRNAs over several days in real time. While a variety of strategies exist to activate gene silencing after the deployment of caged siRNAs, to the best of our knowledge, we believe that this is the first *in vivo* example of an siRNA that can *reversibly* control gene silencing through optical means.

## Supporting Information Available

Experimental details for RNA synthesis, embryo care, injection, and light treatments (PDF)
