# Supplementary material for: In Vivo Injection of Reversible Optically Controlled Short Interfering RNA into Japanese Medaka Embryos (Oryzias latipes) to Regulate Gene Silencing
Source: ACS Chem Biol. 2024 Aug 20;19(9):1904–9. doi: 10.1021/acschembio.4c00290 (PMC11421425; doi:10.1021/acschembio.4c00290)
Supplement: Supplementary file 1 — cb4c00290_si_001.pdf [file cb4c00290_si_001.pdf]

In vivo Injection of Reversible Optically Controlled Short Interfering RNA into Japanese Medaka Embryos (*Oryzias latipes*) to Regulate Gene Silencing

Makenzie Mateus, Matthew L. Hammill, Denina Simmons and Jean-Paul Desaulniers*

Faculty of Science, Ontario Tech University, 2000 Simcoe Street North, Oshawa ON L1G 0C5 Canada

Supporting Information

Experimental procedure for the synthesis and characterization of RNA

Oligonucleotide synthesis and purification S2
LC/MS characterization S3
HPLC characterization S3
Duplex Characterization - CD characterization experiments........S3

Experimental procedure for embryo care, injection, and light treatments

Medaka maintenance and embryo rearing S3
Injection of siRNA into single cell embryo........S4
No light exposure (Dark) experiment........S4
Green light exposure (Azo Green) experiment........S5
Green to Blue light exposure (Azo Green/Blue @72) experiment........S5
Blue to Green light exposure (Azo Blue/Green @72) experiment........S5
eGFP fluorescence measurements........S5
Statistical analysis S5

Figures and Tables

Table S-1: Sequences of anti-luciferase siRNAs, predicted and recorded mass S6
Figure S-1: HPLC chromatogram of antisense strand of F-siRNA-G1 ....S7
Figure S-2: CD spectra of Wt, F-siRNA-G1, and SCR-F-siRNA-G2 ....S8

RNA synthesis and purification

The standard β-cyanoethyl 2'-*O*-TBDMS protected phosphoramidites, reagents and solid supports used to synthesize the Wt and F-azobenzene modified antisense strands were purchased from ChemGenes corporation and Glen Research. The complimentary sense strands were purchased from Integrated DNA Technologies. Strands were synthesized with a sequence intended to target the eGFP gene shown in **Table 1.** Standard phosphoramidites were dissolved to a concentration of 0.10 M in anhydrous acetonitrile while synthesized azobenzene amidites were dissolved in a mixture (anhydrous acetonitrile / THF = 75%:25%) to a concentration of 0.10 M. Phosphoramidite coupling cycle reagents include acetic anhydride/pyridine/THF (Cap A), 16% N-methylimidazole in THF (Cap B), 0.25 M 5-ethylthio tetrazole in ACN (activator), 0.02 M iodine/pyridine/H_2O/THF (oxidation solution), and 3% trichloroacetic acid/dichloromethane. Each sequence was synthesized on a 1.00 μM dT solid support column running for a coupling time of 999 seconds using an Applied Biosystems 394 DNA/RNA synthesizer operating at a 1.00 μM cycle kept under argon at 55 psi. Each cycle consists of five steps: an acid catalyzed deprotection of DMT in which a primary alcohol reacts with the 2' phosphoramidite of the next base in the sequence. An activation step where diisopropylamine is displaced from the phosphoramidite by reacting with ethylthiotetrazole. A coupling step due to the exposed primary alcohol attacking the phosphoramidite linking the bases together. An oxidation step where phosphite is converted to phosphate through water, pyridine, and iodine. The cycle will then either continue coupling the next base or when fully synthesized oligonucleotides can be cleaved from the solid support. Once each cycle was completed the columns were detached off the synthesizer and stored at 4 °C after being placed in a sealed tube. Using 1 ml of EMAM (methylamine 40% in H_2O / methylamine 33% in ethanol = 1:1 (Sigma-Aldrich)) oligonucleotides were cleaved from their respective columns leaving the solution in contact with the pore glass for 1 hour. To fully deprotect the bases the columns were left to incubate in EMAM solution overnight. The next morning the samples were concentrated under reduced pressure and resuspended carefully in DMSO:3HF/TEA (100 μL:125 μL). To remove the 2'-*O*-TDBMS protecting groups the solution was incubated at 65 °C for 3 hours. To desalt the RNA, the strands were placed in dry ice for 1 hour after being precipitated in ethanol and centrifuged at 4 °C for 30 minutes. Oligonucleotides were then run through Millipore Amicon Ultra 3000 MW cellulose. Equal concentrations of sense and antisense RNA were annealed by heating complimentary strands to 95 °C for 2 minutes in binding buffer (75.0 mM KCl, 50.0 mM TrisHCl, 3.00 mM $MgCl_2$, pH 8.30) and allowing the solution to cool to room temperature.

LC/MS characterization

LC/MS chromatograms were obtained on an Agilent 6545 QTOF-MS with Agilent 1260 Infinity Binary Pump HPLC using a ZORBAX Eclipse Plus C18 2.1x100mm 1.8-Micron Agilent column and a mobile phase of 5 mM ammonium acetate buffer (pH 7)/acetonitrile (95:5). Sample preparation included diluting oligonucleotides to a concentration of 0.01 O. D/μL. An injection volume of 20 μL/ sample was used. Data was analyzed using MassHunter Workstation Qualitative Analysis Software (Qual. 10.0).

HPLC characterization

Using a Waters 1525 binary HPLC pump with a waters 2489 UV/Vis detector and C18 4.6 mm x 150 mm reverse phase column, HPLC chromatograms were taken. Empower 3 software was for analysis of scans. An injection volume of 100 μL/ sample was used while running the column at 5% acetonitrile in 95% 0.1 M TEAA (Triethylamine-Acetic Acid) buffer up to 100% acetonitrile over 30 min.

Duplex characterization – Circular Dichroism experiments

CD spectra were calculated by averaging three replicates on Jasco's Spectra Manager version 2 software, ran on a Jasco J-815 CD equipped with a temperature controller. Equal concentrations of sense and antisense strands were annealed in 999 μL of CD sodium phosphate buffer (90.0 mM NaCl, 10.0 mM Na_2HPO_4, 1.00 mM EDTA, pH 7.00) by heating complimentary strands to 95 °C for 2 minutes and allowing the solution to cool to room temperature. When annealed 500 μL of this solution was pipetted into a CD cuvette and ran on the CD with a blank to measure against the baseline of the buffer. Measurements were recorded in triplicate with duplex's being scanned at 25 °C from 200-500 nm at a scanning rate of 20.0 nm/min and a 0.20 nm resolution.

Medaka maintenance and embryo rearing

Fish were housed in the Aquatic Omics lab with a maximum density of 2.3 cm of fish length per liter of water. To ensure genetic variety between test eggs two medaka rearing tanks were used. They were fed live brine shrimp twice daily (once in the morning, once in the afternoon) and flakes once per day (at Noon) during the breeding period. Currently, the Aquatic Omics lab at Ontario Tech University houses the wild type (wt) and d-rR-Tg(beta-actin-loxP-GFP) strains that were used in this experiment purchased from NBRP in Japan. This transgenic strain allows for the silencing of eGFP to be easily and efficiently examined after injection with siRNAs in single cell embryos. It is important to collect and inject the eggs during this period as it ensures proper siRNA distribution to each rapidly dividing cell. However, before injecting, fertilized eggs must be collected. Stage 1 embryos were obtained by placing 1 male in a tank for every one 1 female and controlling for consistent photoperiods of 14 hours of light and 10 hours of dark to simulate spring breeding conditions for optimal production. Fish breeding typically occurs immediately after light stimulation at 8: 00 am; however, it can occur up to two hours post onset. As such, the fish were monitored during this time period. After observing typical courtship behaviour which include a male sporadically chasing a female and then performing quick circling movements beneath her. If accepted the male will wrap its anal fin at the posterior of the female and she produces the oocytes which remain attached to her body during fertilization. After fertilization, the embryos can remain on the mother for a varied amount of time, approximately 24 to 48. Eggs were obtained no later than 10 minutes post fertilization to ensure they were in the single cell stage (stage 1) for injection. A net was used to catch the female medaka and hold her close to the surface of the water while retrieving the fertilized embryos. Using a 50 ml pipette and an aspirator, the eggs were carefully and gently removed from the females' abdomens while remaining in the water to reduce stress. If the eggs could not be removed this way, the female was briefly taken out of the water and the eggs were removed from her abdomen with a finger or thumb. Once collected, the eggs were

stored in a petri dish with tank water. The filaments on the embryos were removed using tweezers. The embryos were then gently rolled over P1200 sandpaper to remove any remaining debris and placed back into petri dish containing a mixture of 50% tank water and 50% rearing solution (methylene blue, NaCl, $MgS0_4$, $CaCl_2$, and KCl dissolved in MilliQ water). Finally, to ensure the eggs remained at the one cell stage for as long as possible, the petri dish was kept on ice until injection.

Injection of siRNAs into medaka embryo

Special needles were prepared from aluminosilicate glass capillaries (outside diameter 1.00 mm inside diameter 0.64 mm) using a P-97 Micropipette Puller from Sutter Instruments under these settings: Pressure = 833, Heat = 300. Pull = 45, Velocity = 70, and Delay = 120 seconds. The pulled needles were snipped at the tip under a stereomicroscope using a razor blade. The needle was then backfilled with mineral oil and placed on the Drummond NanoJect III microinjector (Broomall, PA, USA). Using a HAMILTON 10 µL needle 0.5 µL of siRNA is placed on as fresh sheet of parafilm and then lining the tip of the needle with the spot of liquid the Nanoinjector is filled with injectate. It is important to perform this action slowly as any air bubbles introduced into the injector needle may cause problems such as a loss of suction when injecting. Using a plastic pipette all the collected embryos were placed at the north side of a glass petri dish rolling each individual egg to the center of the dish when ready to inject. Eggs were manipulated using tweezers to allow the single cell of the embryo to face the injector needle perpendicularly. Finally, carefully using the right-hand micro-adjuster, the needle was inserted into the cell and the siRNA injected. Injection amounts were carried out at 8ng and 4ng of siRNA/ egg for WT/ F-siRNA-G1 and 8ng /egg for SCR-F-siRNA-G2. After injections any non-viable embryos were removed to not interfere with the results.

Light activation and inactivation of photoswitchable azobenzene siRNA

No light exposure (Dark) experiment

Immediately after being injected with F-siRNAs, individual embryos were transferred from a petri dish to separate wells (1 embryo per well) of a Costar 96 Well Optical Btm PolymerBase black plate by Thermofisher Scientific using a plastic pipette. Each well with an embryo was filled completely with embryo rearing solution. The plate had no light exposed to it other than natural light to determine if F-siRNA-G1 would silence as well as Wt-siRNA-G1. The eGFP of each egg was measured as stated below in section 2.9 every 24 hours for 10 days. If during this period, an embryo became non-viable the egg was removed and the previous measurements for that well were deleted from the data set for each of the light experiments.

Green light exposure (Azo Green) experiment

Immediately after being injected with F-siRNAs, individual embryos were placed into the wells of a black 96 well plate (one embryo per well) and exposed to green wavelength light (530 nm) using a 4.0-Watt discrete green LED wavelength 530 nm (LEDsupply) for 1 hour to inactivate the siRNA to the *cis* conformer. The embryos were approximately 14 cm away from the LED light. Due to

being exposed to natural light during each fluorescence reading every 24 hours post injection (hpi) the embryos were re-exposed to the same green light.

Green to Blue light exposure (Azo Green/Blue @72) experiment

Immediately after being injected with F-siRNAs, individual embryos were placed in the wells of a black 96 well plate and exposed to green wavelength light (530 nm) for 1 hour to inactivate the siRNA to the *cis* conformer. After each eGFP reading every 24 hpi the embryos were re-exposed to the same green wavelength light until 72 hpi when the embryos were then exposed to a blue light every 24 hours using a 4.0- Watt blue LED (LEDsupply) 470 nm (+/- 5 nm) wavelength to reactivate the siRNA to the *trans* conformer.

Blue to Green light exposure (Azo Blue/Green @72) experiment

Immediately after being injected with F-siRNAs, individual embryos were placed in the wells of a black 96 well plate and exposed to blue wavelength light (470 nm) for 1 hour to activate the siRNA to the *trans* conformer. After each eGFP reading every 24 hpi the embryos were re-exposed to the same blue wavelength light until 72 hpi when the embryos were then exposed to a green light every 24 hours (530 nm) inactivating the siRNA to the *cis* conformer.

eGFP fluorescence measurements

Measurements of fluorescence were taken using a BioTek CytationGen5 plate reader by Agilent with expression being quantified in relative fluorescence units (RFU). Each embryo had to be centered in a 96 well plate using a tweezer to detect optimal fluorescence with a read height of 8.00 nm. The detection method was set to absorbance, read type was set to endpoint kinetic, and optic was set on monochromators. The exposure was kept to a minimum for each run to ensure a baseline between embryos and the specific fluorophore used was eGFP. To eliminate autofluorescence, unmodified wild type medaka embryos fluorescence was measured at each time point and subtracted.

Statistical analysis

Statistical analysis was performed using GraphPad Prism 10. Data is represented at mean ± s.d. Values in figures are reported as eGFP fluorescence as a % of our control denoted as a red dotted line (noninjected embryos). A Two-way ANOVA with a Tukey's post hoc test was used to compare each light treatment to one another with an emphasis on comparing them back to the control within the same time point. An additional repeated measures Two-way ANOVA with a post hoc Tukey's test was conducted to compare each light treatment to the same light treatment at a different time point.

Strand	siRNA sequence	Target	Predicted mass of antisense	Actual mass [b]
Wt-siRNA-G1	3’-dtdt GUU CGA CUG GGA CUU CAA G-5’(s)<br>5’-CAA GCU GAC CCU GAA GUU Cdtdt-3’(as)	eGFP	--	--
F-siRNA-G1	3’-dtdt GUU CGA CUG GGA CUU CAA G-5’(s)<br>5’-CAA GCU GAC AzoU GAA GUU Cdtdt-3’(as)	eGFP	6621.7	6621.8
SCR-F-siRNA-G2	3’-dtdt GUA CGU CGU GAG CUU ACA G-5’(s)<br>5’-CAU GCA GCA AzoC GAA UGU Cdtdt-3’(as)	N/A	6618.9	6619.7

Table S-1 Oligonucleotide strands that were tested on transgenic Japanese Medaka embryos expressing eGFP. WT-siRNA-G1 reflects the full unmodified sequence used to silence eGFP while F-siRNA-G1 represents the tetrafluorinated azobenzene modified siRNA. SCR denotation represents a scrambled sequence used to act as a control.
*Incorporation of the azobenzene modification denoted as Azo at site in strand.

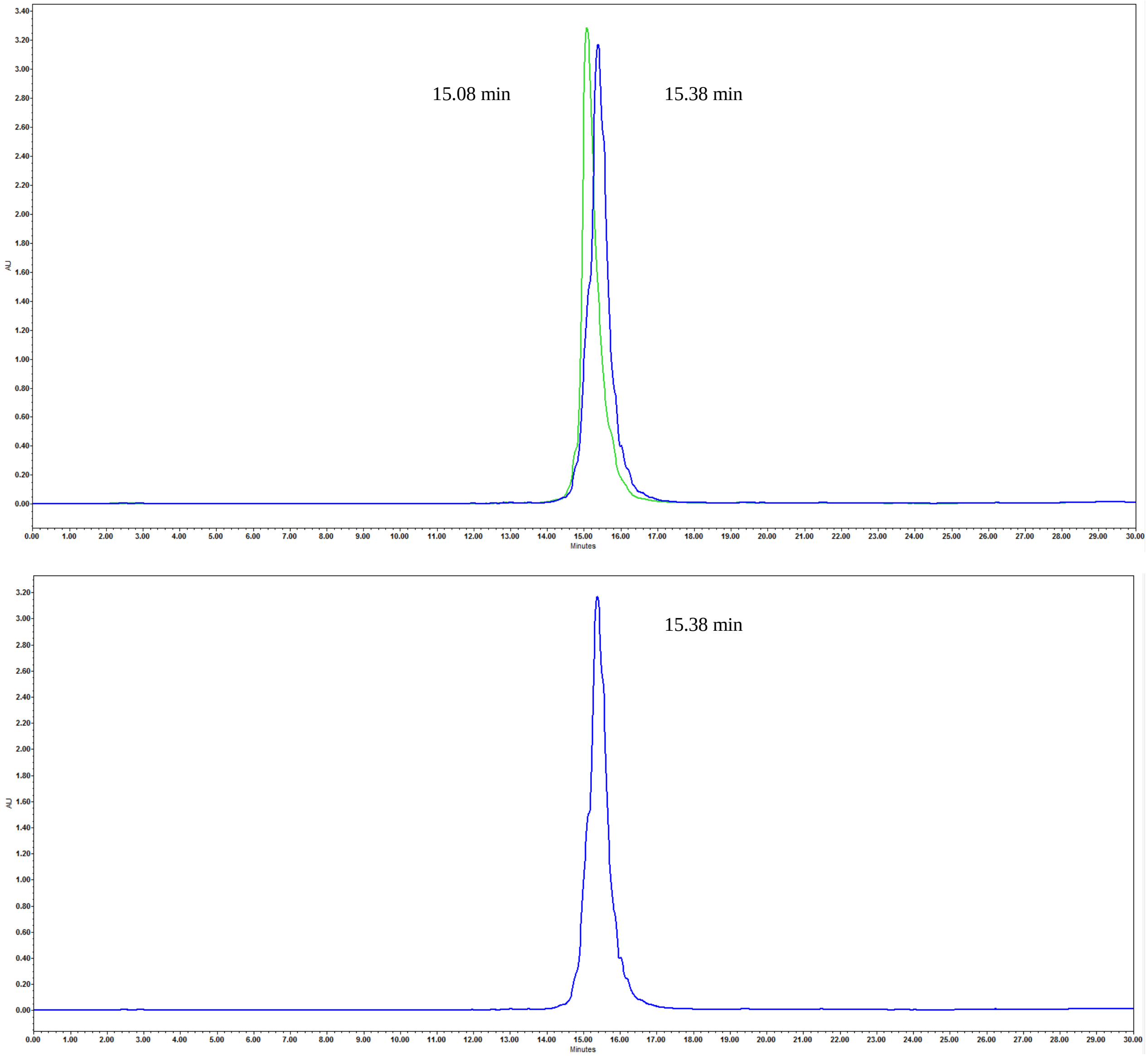

Figure S-1. HPLC chromatogram for the antisense strand of F-siRNA-G1 in the trans conformer (blue) and the cis conformer (green). Conditions were 5% arenonitrile 0.1 M TEAA (Triethylamine-Acetic Acid) buffer up to 100% acetonitrile over 30 min. To ensure the strand was in the trans conformer the experiment was conducted in the dark with only 470nm blue light being exposed to the F-siRNA-G1 strand. This was repeated for the cis conformer using 530nm green light. Spectra were processed using the Empower 3 software.

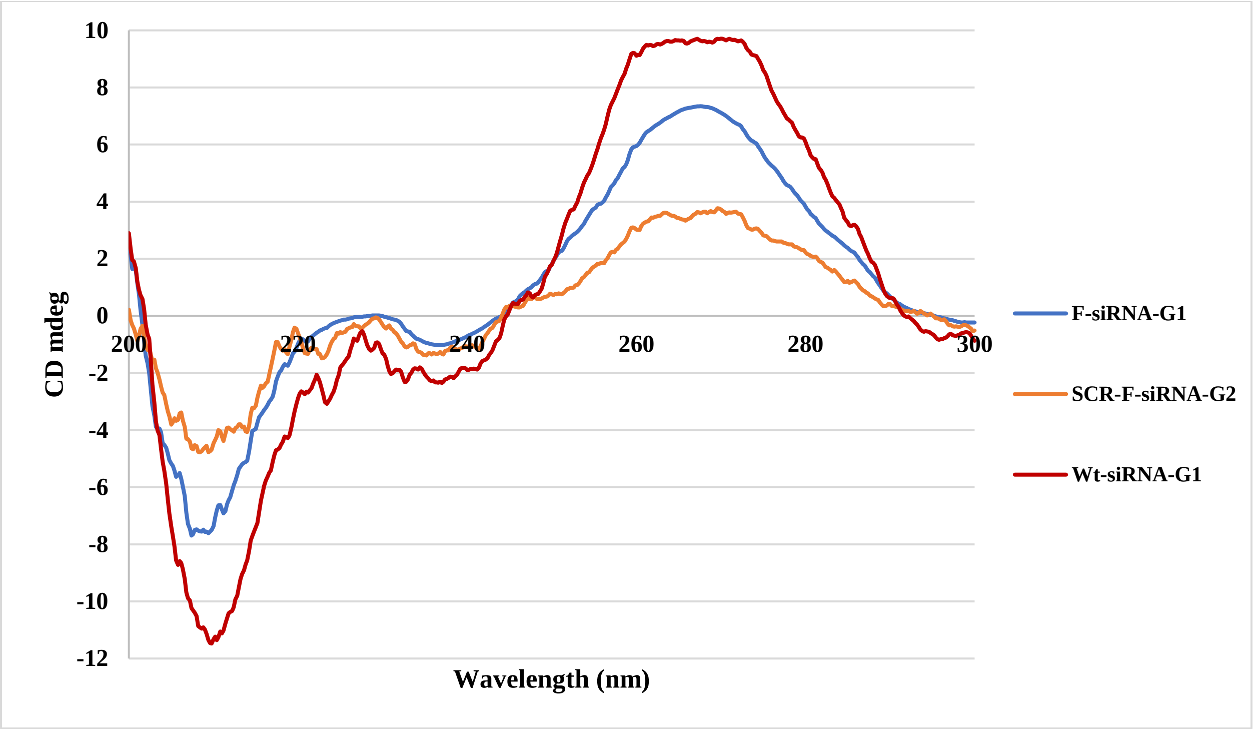

Figure S-2. CD spectra of Wt-siRNA-G1, F-siRNA-G1, and SCR-F-siRNA-G2. The siRNAs (10 μM/duplex) were suspended in 500 μL of a sodium phosphate buffer (90.0 mM NaCl, 10.0 mM Na2HPO4, 1.00 mM EDTA, pH 7.00) and scanned from 200-300 nm at 25 °C with a scanning rate of 20.0 nm/min and a 0.20 nm resolution. All scans were performed in triplicate and averaged using Jasco's Spectra Manager version 2.
